# HAdV-C6 Is a More Relevant Challenge Virus than HAdV-C5 for Testing Antiviral Drugs with the Immunosuppressed Syrian Hamster Model

**DOI:** 10.3390/v9060147

**Published:** 2017-06-13

**Authors:** Karoly Toth, Jacqueline F. Spencer, Baoling Ying, Ann E. Tollefson, William S. M. Wold

**Affiliations:** Department of Molecular Microbiology and Immunology, Saint Louis University, St. Louis, MO 63104, USA; toth@slu.edu (K.T.); spencejf@slu.edu (J.F.S.); yingb@slu.edu (B.Y.); tollefae@slu.edu (A.E.T.)

**Keywords:** adenovirus, hamster, animal model, antiviral

## Abstract

Adenovirus infections of immunocompromised patients can cause a severe multi-organ disease that often results in the patients’ death. Presently, there are no drugs specifically approved to treat adenovirus infections, and clinicians resort to the off-label use of antivirals that are approved to treat other DNA virus infections, most frequently cidofovir (CDV). CDV, however, has considerable nephrotoxicity, thus it is recommended only for the most severe cases of adenovirus infections. To facilitate the development of effective, non-toxic antivirals against adenovirus, we have developed a permissive animal model based on the Syrian hamster that can be used to test the efficacy of antiviral compounds. Here, we show that in the hamster model, HAdV-C6 is a more useful challenge virus than the previously described HAdV-C5, because it is filtered out by tissue macrophages to a lesser extent. HAdV-C6 has a 10-fold lower LD50 in hamsters than HAdV-C5 and the pathology is caused by virus replication to a larger extent. We show that valganciclovir (VGCV), a drug that was shown to be active against intravenous HAdV-C5 infection previously, is efficacious against HAdV-C6 when administered either prophylactically or therapeutically. Further, we show for the first time that VGCV, and to a lesser extent CDV, can be used to treat respiratory adenovirus infections in the hamster model. These results extend the utility of the hamster model, and demonstrate the efficacy of two drugs available for clinicians to treat adenovirus infections.

## 1. Introduction

Adenoviruses (Ads) are viruses with a non-enveloped icosahedral capsid and a double-stranded DNA genome. Human Ads are classified into species A to G that consist of over 70 types (e.g., human species C type 5 adenovirus [HAdV-C5; referred to as Ad5 in this paper]) (for a review on Ad biology, see [1,2]). Ads are widespread and generally cause mild infections in immunocompetent adults that resolve without serious consequences (for a review of Ad epidemiology and pathology, see [3]). The symptoms of Ad infection depend on the type that caused the infection; e.g., types belonging to species B, C, and E usually cause respiratory disease, species F Ads cause enteric infection, and some of the species D viruses are implicated in ocular illnesses. Infection with Ads usually happens in early childhood (ca. 60% of patients with Ad infection are 5 years old or younger [4]). After recovering from infection, the host acquires long-term immunity to that specific type.

In some instances, Ad infection of healthy adults can cause serious illness; certain species D Ads cause epidemic keratoconjunctivitis, which can result even in blindness. Infection with some species B Ads is endemic among military recruits, causing severe acute respiratory disease (for Ad pathology, see [3,5,6]). However, it is immunocompromised patients who suffer the most severe consequences of Ad infection. The defective immune systems of these patients cannot eliminate the virus, and thus the infection can lead to a serious, often life-threatening multi-organ illness. Pediatric allogeneic hematopoietic stem cell transplant (HSCT) patients are especially threatened. The incidence of Ad infection is between 3% and 47% for these patients, and the mortality rate is very high [3,5,7,8,9,10,11,12,13]; approaching 100% for patients with escalating viremia [11].

Thus far, the available countermeasures are inadequate; currently, there is no specific FDA-approved drug available to treat Ad infections. Cidofovir (CDV), a drug approved to treat cytomegalovirus (CMV) retinitis, is often used to treat serious cases of Ad infection. Animal experiments and case reports suggest that CDV is reasonably effective against the virus; however, CDV is kidney toxic, and thus its use is limited to the most serious cases [14]. Ribavirin and to a lesser extent ganciclovir (GCV) have also been used (for reviews, see [3,5,6,7,8,9,10,11,15,16]). Brincidofovir (BCV, hexadecyloxypropyl-cidofovir, formerly known as CMX001), a less toxic derivative of CDV, showed good efficacy in vitro and in animal studies, but the ongoing clinical trials must be completed before approval [17,18,19,20,21,22]. Clearly, further research is needed to develop anti-adenoviral drugs. 

The development of such drugs was hampered by the lack of an adequate animal model to test their efficacy. To address this deficiency, we developed an animal model based on the permissive Syrian hamsters (reviewed in [23]), which is amenable for in vivo studies to test the efficacy of anti-adenoviral compounds [24,25,26,27]. To mimic disseminated Ad infection in immunocompromised patients, the animals are immunosuppressed by cyclophosphamide [24] and then are infected intravenously (i.v.) with Ad5. This route of challenge leads to rapid Ad replication in multiple organs, most significantly in the liver [28,29,30]. The replication of the virus will lead to high virus burden and hepatocellular necrosis, and the animals will present with quantifiable pathology (mortality, body weight loss, elevation of serum transaminase levels, microscopic pathology) [30,31], similarly to immunocompromised patients with advanced disseminated multi-organ Ad infections. We have demonstrated that the symptoms of Ad5 infection in hamsters can be mitigated or even reversed with the use of BCV [24,27], GCV [26], valganciclovir (VGCV) [25], or CDV [27]. 

Previously, we have shown that Ad6 (HAdV-C6), another species C human Ad, is capable of replicating in Syrian hamsters, and that it is more pathogenic than Ad5 [29]. HAdV-C5 and C6 are 99% identical at the nucleotide level. However, there are important differences in the length of fiber shaft and the sequence of the hexon hypervariable regions that may influence the tropism and resulting pathogenicity of these viruses [32]. Here, we show that VGCV, and to a lesser extent CDV, are capable of inhibiting Ad6 replication and mitigating Ad6-induced pathology. For the first time, we also show that a drug is effective against respiratory challenge with Ad.

## 2. Materials and Methods

### 2.1. Cells and Viruses

A549 human lung adenocarcinoma cells were purchased from the American Type Culture Collection (ATCC) (Manassas, VA, USA), while HEK293 human embryonic kidney cells were purchased from Microbix (Mississauga, ON, Canada). Both cell lines were cultured in Dulbecco’s modified Eagle’s medium (Sigma-Aldrich, St Louis, MO, USA) with 10% fetal bovine serum (FBS) at 37 °C. The Ad5 isolate we used is named Ad5 *wt500*, and it was derived by plaque purification by our laboratory from a wild-type Ad5 stock purchased from ATCC. A human Ad6 isolate (Tonsil 99; VR-6) was purchased from ATCC and cultured and purified as described previously [33]. The titer of the virus stocks was determined by plaque assay [33]. 

### 2.2. Antiviral Compounds

VGCV (batch 20130325) was purchased from 2A Pharmachem (Lisle, IL, USA), and dissolved in water at 20 mg/mL for the animal dose of 200 mg/kg. CDV was obtained from the National Institute of Allergy and Infectious Diseases (NIAID) and dissolved in phosphate-buffered saline (PBS) at 3.7 mg/mL and 2 mg/mL for the 37 mg/kg and 20 mg/kg doses, respectively.

### 2.3. Syrian Hamsters

Syrian hamsters (*Mesocricetus auratus*) were purchased from Envigo (Indianapolis, IN, USA) at approximately 100 g body weight. Female animals were used for all experiments except the repeat of the intranasal (i.n.) challenge, VGCV treatment experiment. All studies were approved by the Institutional Animal Care and Use Committee of Saint Louis University and were conducted according to federal and institutional regulations. 

### 2.4. Infection of Hamsters with Adenovirus

The animals were immunosuppressed using cyclophosphamide (CP) as described before [24]. CP was administered intraperitoneally (i.p.) at a dose of 140 mg/kg, and then at a dose of 100 mg/kg twice weekly for the duration of the study. After three injections of CP, Ad5 or Ad6 was injected i.v. via the jugular vein at a dose of 2 × 10^11^ plaque-forming units (PFU)/kg for Ad5 or 2 × 10^10^ PFU/kg for Ad6 in 200 μL of PBS under ketamine/xylazine anesthesia. For the i.n. challenge, animals were anesthetized with isoflurane, and 1 × 10^10^ PFU/kg of Ad6 in 100 μL of PBS was pipetted into the nostrils. For both routes of administration, control animals received PBS.

### 2.5. Treatment with Antiviral Drugs

VGCV was administered through oral gavage (p.o.), starting 12 h prior to (prophylactic studies) or 1 day after (therapeutic study) Ad6 injection at the dose of 200 mg/kg, and then continued twice daily (b.i.d.) using the same dose. Control animals were gavaged with equal volume of water. CDV was injected i.p. at an initial dose of 37 mg/kg, and then continued three times weekly at the dose of 20 mg/kg. Control animals were injected with PBS.

### 2.6. Treatment Groups and Endpoints

For all experiments, there were groups infected with Ad5 or Ad6 that were either left untreated or were treated with VGCV or CDV. Uninfected (injected with virus vehicle only) groups that were treated with a drug or drug vehicle served as controls. The animals were randomized by body weight before the start of the study. All hamsters were observed and weighed daily. For the prophylactic studies, each group consisted of 15 hamsters. Five hamsters of each group were designated to be sacrificed at 5 days (for the i.v. challenge) or at 3 days (for the i.n. challenge) post Ad challenge. At necropsy, liver, lungs, and kidney (for the i.v. challenge) or lungs only (for the i.n. challenge) were collected. The virus was extracted from the collected organs and was quantified by 50% Tissue Culture Infectious Dose (TCID_50_) assay in HEK-293 cells [24]. A portion of collected tissues was preserved in formalin for histopathology. Serum was collected and assayed for liver transaminase levels (i.v. challenge only). The remaining 10 hamsters in each group were used for a survival study. Besides animals judged moribund by observation, we sacrificed all hamsters that lost more than 20% of their original body weight. The surviving hamsters were sacrificed at 14 days (i.v. experiment) or at 7 or 10 days (i.n. experiment) post challenge. For the therapeutic study, there were 10 hamsters in each group, and the study was conducted as a survival experiment, as described above.

### 2.7. Statistics

Statistical analysis was performed using GraphPad Prism 7 (GraphPad Software). Two-way ANOVA was used to compare body weight changes. For serum transaminase levels and virus burden in the liver, the overall effect was calculated using the Kruskall–Wallis test, and pair-wise comparison was done using the Mann–Whitney U-test. The log rank test was used to analyze survival data where *p* ≤ 0.05 was considered significant.

## 3. Results

### 3.1. Prophylactic Treatment with VGCV Is More Efficacious against Intravenous Challenge with Ad6 than against Ad5 despite Ad6 Being More Pathogenic than Ad5 in Immunosuppressed Syrian Hamsters

To investigate whether VGCV is active against Ad6 infection, we infected immunosuppressed hamsters with Ad5 or Ad6. To equalize the pathogenicity of the two viruses, we administered 10-fold less Ad6 than Ad5 (2 × 10^11^ PFU/kg of Ad5 v. 2 × 10^10^ PFU/kg of Ad6). VGCV was used at a dose and treatment schedule known to be effective against Ad5 (200 mg/kg b.i.d., starting one day before challenge) [25]. 

**Figure 1:**
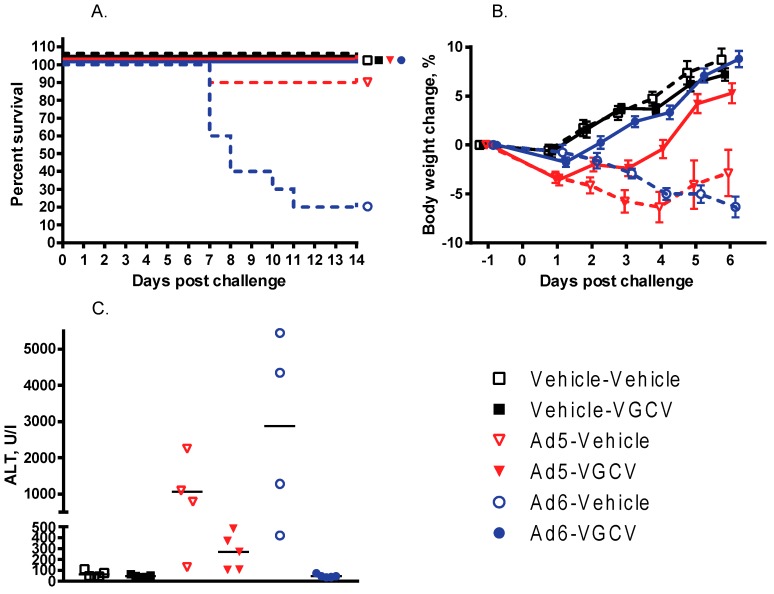
Valganciclovir (VGCV) prevents mortality and mitigates Ad5- and Ad6-induced liver damage in immunosuppressed Syrian hamsters. (**A**) Survival. Ad6-Vehicle v. Ad6-VGCV *p* = 0.0002 (Log rank); (**B**) Body weight changes. Group means are shown only up to 6 days post challenge; after this point, the deaths in the Ad5-Vehicle and Ad6-Vehicle groups would confound group means. The error bars show the standard error of the mean; (**C**) Serum alanine transaminase levels. Each symbol represents the value from an individual animal; the horizontal bar signifies the mean. Ad6-Vehicle v. Ad6-VGCV *p* = 0.0159, Ad5-Vehicle v. Ad5-VGCV *p* = 0.1111 (Mann–Whitney U-test).

There were nine treatment-related deaths in the study, one in the Ad5-infected vehicle-treated group and eight in the Ad6-infected vehicle-treated group (Figure 1A). VGCV treatment prevented mortality in both the Ad5- and Ad6-infected VGCV-treated groups, and no deaths occurred in either of the uninfected groups. Hamsters in both of the Ad-infected untreated groups were losing weight from the onset of the study (Figure 1B). VGCV treatment completely reversed body weight loss for both Ad5- and Ad6-challenged hamsters (Figure 1B). No animals in the uninfected groups lost weight during the study (Figure 1B).

There was a difference in the kinetics of weight loss between the Ad5- and Ad6-injected groups: the Ad5-challenged animals lost weight fast for the first 3 to 5 days of the study, but then the surviving hamsters started recovering (Figure 1B). Conversely, animals in the Ad6-infected vehicle-treated group looked healthier for the first 3 days of the study; however, these animals started losing weight rapidly from day 4 on (Figure 1B).

At necropsy at 5 days post challenge, three of the four Ad6-infected vehicle-treated hamsters and two of the four Ad5-infected vehicle-treated hamsters exhibited liver pathology. One Ad6-challenged animal had red, mottled lungs. No significant findings were noted for the Ad-infected VGCV-treated and uninfected animals. The hamsters sacrificed moribund showed severe liver lesions (yellow, mottled, friable liver; enlarged gall bladder). In addition to the liver lesions, about one-third of the Ad6-infected animals presented with pathology in their lungs (enlarged, firm lungs with dark red to black discoloration) and their kidneys (grossly enlarged, pale kidneys). No such pathology was noted for the Ad5-infected hamsters. At 14 days post challenge, no significant findings were noted for any of the surviving animals. Microscopically, the livers of the Ad-infected vehicle-treated hamsters sacrificed moribund exhibited moderate hepatocellular necrosis and a severe decrease in hepatocellular vacuolization. No such changes were noted for the Ad-infected VGCV-treated animals; for these hamsters, minimal acute inflammation was the only significant histopathological finding. 

To quantify the effect of VGCV on liver damage with Ad6-infected hamsters, serum was collected at necropsy and analyzed for transaminase levels. The serum alanine transaminase (ALT) levels were very high for all the Ad5- and Ad6-infected vehicle-treated hamsters (Figure 1C) with the Ad6-infected animals exhibiting on average three times higher ALT levels than the Ad5-challenged ones. Notably, VGCV completely prevented liver pathology for the Ad6-infected animals; the serum ALT levels were normal for the Ad6-infected VGCV-treated hamsters (Figure 1C). VGCV treatment mitigated liver pathology for Ad5-challenged hamsters as well, although the difference was not statistically significant (Figure 1C). 

**Figure 2:**
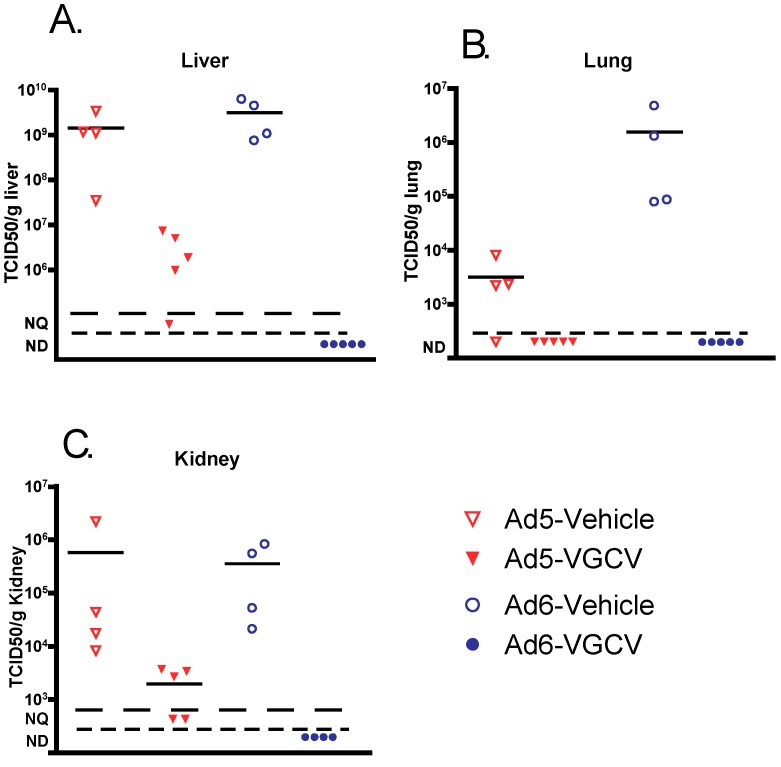
VGCV decreases Ad5 and Ad6 virus burden in the (**A**) liver, (**B**) lungs, and (**C**) kidney of immunosuppressed Syrian hamsters. Each symbol represents the value from an individual animal; the horizontal bar signifies the mean. NQ: not quantifiable; ND: not detectable. Significance was not calculated because of not detectable (not scalar) levels in the VGCV-treated groups.

To determine whether VGCV prevented pathology by inhibiting Ad replication in target organs, liver, lungs, and kidney samples were collected at necropsy and analyzed for infectious virus burden. Vehicle-treated Ad-infected animals had a very high virus burden in all of the tested organs (Figure 2), with the Ad6 burden being generally higher than Ad5. VGCV treatment reduced the virus burden in all three organs for both Ad5- and Ad6-infected hamsters. Remarkably, VGCV reduced the Ad6 virus burden to undetectable levels (Figure 2).

### 3.2. Therapeutic Treatment with VGCV Prevents Ad6 Infection-Induced Mortality and Morbidity in Immunosuppressed Syrian Hamsters

We demonstrated before that VGCV prevents the mortality and morbidity of Ad5-infected hamsters when administered therapeutically, i.e., after virus challenge [25]. To determine whether VGCV is similarly active against Ad6 infection, we infected immunosuppressed hamsters i.v. with 2 × 10^10^ PFU/kg of Ad6, and treated the animals with 200 mg/kg of VGCV starting one day after challenge, and continued the treatment b.i.d. for the duration of the study.

**Figure 3:**
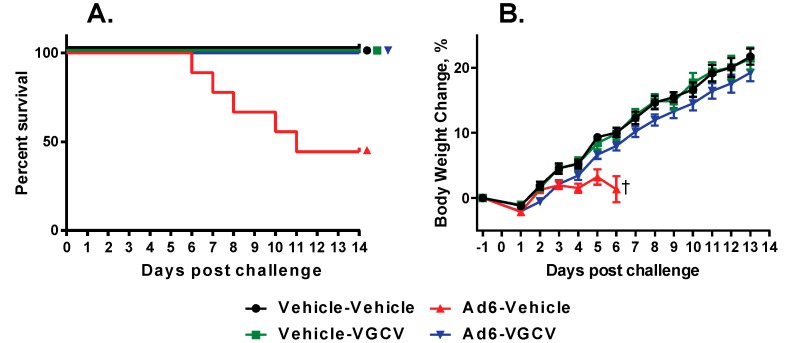
Therapeutically administered VGCV prevents mortality and mitigates Ad6-induced morbidity in immunosuppressed Syrian hamsters. (**A**) Survival. Ad6-Vehicle v. Ad6-VGCV *p* = 0.0067 (Log rank); (**B**) Body weight changes. † Means for the Ad6-Vehicle group are shown only up to 6 days post challenge; after this point, the deaths would confound group means. The error bars show the standard error of the mean.

There were five treatment-related deaths in the study, all in the Ad6-infected vehicle-treated group (Figure 3A). Upon sacrifice, these animals showed liver pathology that is characteristic of disseminated Ad infection in this model. VGCV treatment completely prevented mortality in the Ad6-infected VGCV-treated group. Hamsters in the Ad6-infected untreated group were losing weight from the onset of the study (Figure 3B); VGCV treatment completely reversed this body weight loss (Figure 3B). 

### 3.3. VGCV Suppresses Virus Replication in the Lungs and Mitigates Morbidity after Ad6 Respiratory Infection of Immunosuppressed Female and Male Hamsters

Respiratory infection is one of the main routes by which patients acquire Ads. To test whether prophylactic administration of VGCV can affect the outcome of respiratory Ad6 infection, we tested the efficacy of VGCV against i.n. challenge with the virus. We pipetted the virus dose into the nostrils of anesthetized hamsters, which inhaled the liquid, thus delivering the virus to the lower airways [29]. For the VGCV-treated animals, we used the same drug dose and dosing schedule as for the i.v. experiment. 

**Figure 4:**
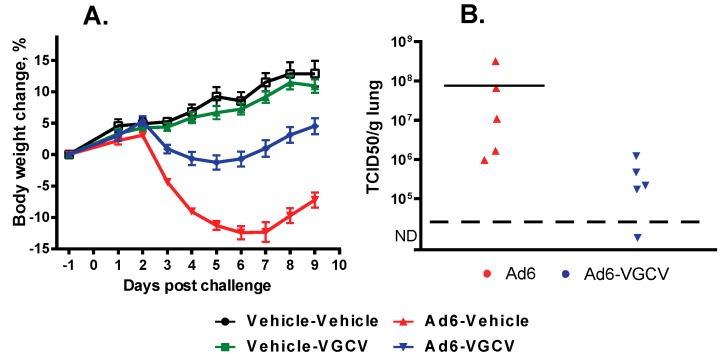
VGCV reverses Ad6-induced morbidity and decreases Ad6 virus burden in the lungs of immunosuppressed female Syrian hamsters. (**A**) Body weight changes. Each symbol represents the group mean; the error bars depict the standard error of the mean. Ad6-Vehicle v. Ad6-VGCV *p* < 0.0001 (two-way ANOVA); (**B**) Virus burden in the lungs. Each symbol represents the value from an individual animal; the horizontal bar signifies the mean. Ad6-Vehicle v. Ad6-VGCV *p* = 0.0159 (for statistical calculation, a value of 1 × 10^3^ PFU/g was assigned to the single “not detectable” sample).

There were no treatment-related deaths in the study. Hamsters in the Ad6-infected groups were losing weight starting at 2 days post challenge (Figure 4A); VGCV treatment mitigated body weight loss (Figure 4A). No body weight loss was seen in the drug-only control group. At the 3 days post challenge sacrifice time point, all five of the sacrificed animals in the Ad6-Vehicle group presented with lung pathology (red mottled discoloration), while only three of the five Ad6-infected VGCV-treated hamsters displayed such pathology. No significant lesions were noted in other organs. At 10 days post challenge, red mottled lungs were observed in six out of 10 Ad6-Vehicle hamsters and three out of 10 Ad6-VGCV-treated hamsters. 

To examine the effect of VGCV on Ad6 replication in the lungs, lung samples were collected at the Day 3 necropsy and were analyzed for infectious virus burden. Vehicle-treated Ad6-infected animals had a very high virus burden in the lungs, while VGCV treatment significantly reduced the virus burden (Figure 4B).

To investigate whether sex differences influenced the anti-adenoviral efficacy of VGCV, we repeated the i.n. challenge experiment using male hamsters. The methodology of the experiment was identical to the one performed with female animals, with the exception that the experiment was terminated at 7 days instead of 10 days post challenge.

**Figure 5:**
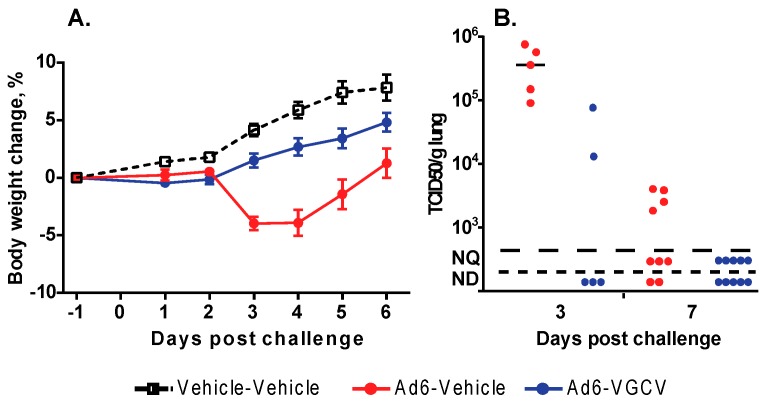
VGCV reverses Ad6-induced morbidity and decreases the Ad6 virus burden in the lungs of immunosuppressed male Syrian hamsters. (**A**) Body weight changes. Each symbol represents the group mean; the error bars depict the standard error of the mean. Ad6-Vehicle v. Ad6-VGCV *p* < 0.0001 (two-way ANOVA); (**B**) Virus burden in the lungs. Each symbol represents the value from an individual animal; the horizontal bar signifies the mean. Statistical significance was not calculated.

Similarly to female hamsters, there were no treatment-related deaths in the study. Hamsters in the Ad6-infected groups lost weight with the same kinetics as Ad6-infected females (Figure 5A), and VGCV treatment had the same mitigating effect (Figure 5A). At 3 days post challenge, three of the five sacrificed animals in the Ad6-Vehicle group presented with lung pathology (red mottled discoloration), while only one of the five Ad6-infected VGCV-treated hamsters displayed such pathology. At 7 days post challenge, all of the A6-infected vehicle-treated hamsters showed lung pathology. The pathology for the Ad6-infected VGCV-treated group was less severe, and no significant findings were noted for two of the 10 hamsters in this group. No significant lesions were noted in other organs for any of the animals at either sacrifice time point. 

To examine the effect of VGCV on Ad6 replication in the lungs of the male hamsters, samples were collected at the Day 3 necropsy and analyzed for infectious virus burden. We found that at 3 and 7 days post challenge, VGCV treatment decreased the virus burden in the lungs compared to untreated animals (Figure 5B).

### 3.4. CDV Mitigates Morbidity of Immunosuppressed Hamsters after Ad6 Respiratory Infection

**Figure 6:**
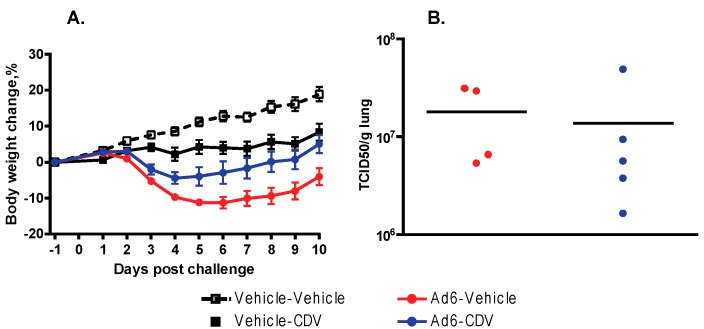
Cidofovir (CDV) mitigates Ad6-induced morbidity. (**A**) Body weight changes. Each symbol represents the group mean; the error bars depict the standard error of the mean. Ad6-Vehicle v. Ad6-CDV *p* < 0.0001 (two-way ANOVA); (**B**) Virus burden in the lungs. Each symbol represents the value from an individual animal; the horizontal bar signifies the mean. Ad6-Vehicle v. Ad6-CDV *p* = 0.5556 (Mann–Whitney U test).

CDV is another compound that is known to be active in the hamster model after i.v. challenge with Ad5 [27]. To assess whether CDV is effective against respiratory challenge with Ad6, we treated i.n.-infected hamsters with the drug. We used the known effective dose (37 mg/kg i.p. followed by 20 mg/kg doses three times weekly), starting the treatment one day before challenge. There were no treatment-related deaths in the study. Hamsters in the Ad6-infected groups were losing weight from the onset of the study (Figure 6A). CDV treatment significantly mitigated body weight loss (Figure 6A); however, it was slightly toxic at the dose used, inasmuch as decreased weight gain was seen in the drug-only control group compared to the vehicle-only group.

At 3 days post challenge, no differences were observed in the extent of lung pathology seen in the Ad6-Vehicle and Ad6-CDV groups; four out of five hamsters had red mottled lungs in both groups. Similarly, no differences were detected between the untreated and treated groups at 10 days post challenge; white foci were found in the lungs in eight of 10 and six of 10 hamsters from the Ad6-Vehicle and Ad6-CDV groups, respectively. 

At 3 days post challenge, we determined the virus burden in the lungs of the Ad6-infected hamsters. Vehicle-treated Ad6-infected animals had a very high virus burden in the lungs, while CDV treatment may have marginally reduced the virus burden (Figure 6B). 

## 4. Discussion

Previously, we demonstrated that VGCV was efficacious against i.v. challenge with Ad5 in the immunosuppressed hamster model. In this present work, we used Ad6 as the challenge virus. Ad6 is sequestered less in tissue macrophages than the closely related Ad5, and with i.v. challenge of hamsters; this decreased uptake by macrophages resulted in increased pathogenicity of Ad6 compared to Ad5 [34]. The LD_50_ of Ad6 is ca. 10-fold lower than that of Ad5, i.e., a 10-fold lower challenge dose is required to elicit the same pathology [29]. VGCV inhibits Ad DNA replication and was shown to be effective against Ad6 in vitro [25]; thus, it was expected to be efficacious against i.v. challenge as well. Indeed, after both prophylactic and therapeutic administration, it inhibited virus replication and prevented mortality and morbidity (Figure 1 and Figure 2). The differences observed in the pathology caused by the two species C Ads confirmed our previous findings. The kinetics of morbidity is also different; Ad5-infected hamsters lost more weight right after infection than Ad6-infected ones; however, morbidity and mortality was greater for the Ad6-infected animals at later time points (Figure 1B). This observation suggests that while some of the pathogenicity by Ad5 might be caused by the input bolus of the virus, for Ad6 (which can be given at a lower dose), most of the pathogenicity is a result of ongoing virus replication. Ad6-infected hamsters developed lesions in the lungs and kidney besides the liver, which is the most prominent site of pathogenicity for Ad5 [29]. Remarkably, VGCV treatment of Ad6-infected animals reduced pathology in the liver, lungs and kidney, and reduced the virus burden in these organs to undetectable levels (Figure 2). This finding opens the possibility of testing the efficacy of anti-Ad compounds in the lungs and the kidney, two additional organs (besides the liver) that are affected in immunocompromised patients with disseminated Ad infections.

Thus far, we and others have shown that several drugs were active against i.v. Ad5 infection [24,25,26,27,35]. However, none of these drugs was effective against respiratory infection with the virus. The presumed reason for this inefficacy is that even in CP-treated animals, much of the pathology after intranasal infection with Ad5 is the result of the immune response to the input virus, inasmuch as we showed that a replication-defective, Ad5-based vector was nearly as pathogenic as Ad5. With the lower LD_50_ of Ad6, we could decrease the input bolus of the challenge virus; and thus more of the observed pathology is caused by actual Ad replication and cytopathic effect. Consistent with this hypothesis, VGCV treatment of hamsters challenged intranasally with Ad6 significantly decreased body weight loss, inhibited the replication of Ad6 in the lungs, and mitigated the pathogenic effects of Ad6. This is the first time that we showed that an antiviral drug inhibits the replication of a human Ad in the lungs of immunosuppressed hamsters, and that this inhibition results in decreased morbidity. 

Sex differences can affect the pharmacokinetics and pharmacodynamics of a drug [36]. However, this is not the case for the anti-adenoviral efficacy of VGCV; we received similar results with female and male animals when treating them after i.n. challenge (Figure 4 and Figure 5). 

We tested CDV, another drug with known activity against i.v. challenge with Ad, in the i.n. challenge model. As with VGCV, we found that prophylactic treatment with CDV mitigated body weight loss and decreased the virus burden in the lungs of Ad6-infected hamsters. However, CDV was less active than VGCV, even though we gave a very high, slightly toxic dose of the drug (Figure 6). While CDV-treatment significantly decreased body weight loss, the decrease in the virus burden in the lungs was not statistically significant, and the treatment did not mitigate the gross pathology observed in the lungs. Although we did not do a direct, head-to-head comparison of the two compounds, these data confirm our previous observation with the i.v. model that VGCV is more active against Ad infection.

These data indicate that while both Ad5 and Ad6 replicate in Syrian hamsters, a 10-fold lower challenge dose of Ad6 than for Ad5 is required to induce the same pathology. This is likely due to the different tropism of Ad5 and Ad6 (lower Ad6 uptake by tissue macrophages). For Ad6, this results in a pathology elicited in large part by virus replication, while the host response to the larger input bolus is a significant part of Ad5 pathology. As most anti-adenoviral compounds are inhibitors of virus replication, we suggest that in the Syrian hamster model, Ad6 challenge is a better way of testing these drugs than Ad5. Further, these data confirm our previous finding that VGCV is very effective against disseminated Ad infection in the Syrian hamster model. Adding to this, we demonstrated that VGCV is efficacious against respiratory Ad infection of hamsters. In these studies, we chose to use VGCV over its parent drug, GCV, because of the less invasive administration route (oral versus intraperitoneal) afforded by the prodrug. We understand that for human patients, GCV is the drug of choice in situations when gastrointestinal co-morbidities preclude oral administration of drugs. We have reported previously that GCV was equally efficacious against HAdV-C5 infection in the hamster model [26]; thus, we believe that our findings described here have a clinical relevance. These data suggest that GCV or VGCV can be used to treat human patients with severe Ad infections.
